# Readiness for Voice Technology in Patients With Cardiovascular Diseases: Cross-Sectional Study

**DOI:** 10.2196/20456

**Published:** 2020-12-17

**Authors:** Małgorzata Kowalska, Aleksandra Gładyś, Barbara Kalańska-Łukasik, Monika Gruz-Kwapisz, Wojciech Wojakowski, Tomasz Jadczyk

**Affiliations:** 1 Department of Epidemiology Faculty of Medical Sciences in Katowice Medical University of Silesia Katowice Poland; 2 Student Scientific Society Department of Epidemiology Faculty of Medical Sciences in Katowice, Medical University of Silesia Katowice Poland; 3 Department of Cardiology and Structural Heart Diseases Medical University of Silesia Katowice Poland; 4 Interventional Cardiac Electrophysiology Group International Clinical Research Center St Anne's University Hospital Brno Brno Czech Republic; 5 Research and Development Division CardioCube Corp Seattle, WA United States

**Keywords:** voice technology, smart speaker, acceptance, telehealth, cardiovascular diseases, chatbot

## Abstract

**Background:**

The clinical application of voice technology provides novel opportunities in the field of telehealth. However, patients’ readiness for this solution has not been investigated among patients with cardiovascular diseases (CVD).

**Objective:**

This paper aims to evaluate patients’ anticipated experiences regarding telemedicine, including voice conversational agents combined with provider-driven support delivered by phone.

**Methods:**

A cross-sectional study enrolled patients with chronic CVD who were surveyed using a validated investigator-designed questionnaire combining 19 questions (eg, demographic data, medical history, preferences for using telehealth services). Prior to the survey, respondents were educated on the telemedicine services presented in the questionnaire while being assisted by a medical doctor. Responses were then collected and analyzed, and multivariate logistic regression was used to identify predictors of willingness to use voice technology.

**Results:**

In total, 249 patients (mean age 65.3, SD 13.8 years; 158 [63.5%] men) completed the questionnaire, which showed good repeatability in the validation procedure. Of the 249 total participants, 209 (83.9%) reported high readiness to receive services allowing for remote contact with a cardiologist (176/249, 70.7%) and telemonitoring of vital signs (168/249, 67.5%). The voice conversational agents combined with provider-driven support delivered by phone were shown to be highly anticipated by patients with CVD. The readiness to use telehealth was statistically higher in people with previous difficulties accessing health care (OR 2.920, 95% CI 1.377-6.192) and was most frequent in city residents and individuals reporting a higher education level. The age and sex of the respondents did not impact the intention to use voice technology (*P*=.20 and *P*=.50, respectively).

**Conclusions:**

Patients with cardiovascular diseases, including both younger and older individuals, declared high readiness for voice technology.

## Introduction

The dynamic growth of telehealth and telemedicine solutions enables patients to access clinical care remotely, which equalizes health care coverage, supports care coordination, and increases the safety of patients and providers through physical distancing. Well-established phone and video consultations [1], along with emerging technologies [2,3], offer a powerful framework for connected health. Among patients with cardiovascular disease (CVD), telemedicine is supported by the European Society of Cardiology and the American Heart Association, which recommend multidisciplinary programs and tele-education for cardiovascular care [4-6]. Moreover, recent meta-analyses concluded that virtual care is more effective for adults with heart failure (HF) compared with standard care, particularly in reducing all-cause mortality, cardiac mortality, and hospitalization rates [7,8]. However, the effectiveness of telehealth programs depends on patient engagement and willingness to use the services [9,10]. Thus, it is crucial to understand patients' expectations and consider them while planning and developing novel telemedical strategies.

The latest advances in the field of artificial intelligence (AI) and natural language understanding have paved a way for the wide application of voice technology (VT) in health care through smart speakers and mobile apps. First real-world implementations have confirmed that AI conversational agents have the potential to support clinical care and optimize workflows [3,11-13]. Nationwide Children’s Hospital (in Columbus, Ohio) implemented voice technology for pediatric medicine [11], while Mayo Clinic developed Amazon’s Alexa software to automatically answer COVID-19–related questions [12]. Furthermore, CardioCube software (CardioCube Corp.) deployed on Amazon Alexa was shown to be functional for paperless medical history collection from patients with CVD [3] as well as for long-term remote follow-up of individuals with HF at home [13]. To evaluate prevailing perceptions and expectations, our study analyzed the opinions of and readiness for VT-based telehealth solutions among patients with CVD.

## Methods

### Study Design

The cross-sectional study was based on the investigator-designed and validated 19-item questionnaire used to recognize factors influencing readiness for telemedicine among individuals with CVD. The methodology included the (1) preparation of the questionnaire (Multimedia Appendix 1), (2) assessment of the questionnaire’s repeatability (questionnaire validation process), and (3) evaluation of anticipated patient experiences regarding telemedical solutions using the previously validated questionnaire, which was provided to the total studied population.

This study was performed at the Department of Cardiology and Structural Heart Diseases, Medical University of Silesia in Katowice, Poland, which hospitalizes approximately 2700 to 2800 individuals per year. The ethics approval for the study was received from the Bioethical Committee of the Medical University of Silesia in Katowice (approval number KNW/0022/KB1/160/1617) on February 3, 2017.

### Questionnaire Validation

The original questionnaire included 19 questions concerning demographic data (sex, age, place of residence, educational level, occupational activity); health status data, such as previously diagnosed diseases, history of cardiovascular treatments, and previous hospitalization; and information about the current form of follow-up contact with a cardiologist. Key questions were related to the patient’s opinions and personal preferences regarding the application of telemedicine for remote contact with a medical doctor. To ensure validity of the questionnaire, the same 30 patients answered the questionnaire twice within the 2- to 3-day interval. The median age of the participants was 65 years. Most of the respondents were men (21/30, 70%), lived in the city (26/30, 87%), had a higher level of education (12/30, 40%), and reported living with another family member (23/30, 77%). To assess the reliability of the questionnaire, the Cohen statistic and interclass correlation coefficient were calculated with simultaneous assessment of the percentage of the repeatability of responses for the key questions of the questionnaire. By the applicable principles, the mean, good, and very good compliance of the Cohen values are represented by the values of 0.41 to 0.60, 0.61 to 0.80, and 0.81 to 1.00, respectively [14]. The key questions had very high repeatability, ranging from 80% to 100%. Detailed results of the validation procedure are presented in Multimedia Appendix 2. As confirmed by the research tool reliability, study participants were surveyed using a validated author-created questionnaire.

### Cross-Sectional Study

Between March 2019 and January 2020, each of the patients admitted to the Department of Cardiology was invited to participate in the questionnaire survey. Written consent to participate in the study was obtained from 249 patients (participation rate of 9%). The participants completed the questionnaires while assisted by the medical doctor, who explained the meaning and potential clinical application of the telemedical solutions presented in the survey, assuring that responders understood the questions (Multimedia Appendix 3).

### Statistical Analysis

Statistical analysis was undertaken using the Statistica 13.0 package (Dell Software). The missing values were removed pairwise from further statistical analysis. The measures of central tendency (median, quartile) and dispersion (interquartile range) were applied in the statistical description of quantitative variables; their distribution was verified using the Shapiro-Wilk test. Qualitative variables were presented using frequency and percentage. Differences between groups were tested using the chi-square or Fisher test. In all analyses, *P* values below .05 were considered statistically significant. Finally, the relevant relationships between particular variables were verified using multivariable analysis (logistic regression models with Hooke-Jeeves and quasi-Newton estimation). The null hypothesis in the adopted model was that patients’ readiness to use telemedicine solutions in general and to use a voice conversational agent specifically are not related to independent parameters, such as female sex, age, place of residence being in the city, primary level of education, living with family, and previous difficulties with medical care. Only the statistically significant variables obtained in bivariate analyses were included in the model. Additionally, a chi-square test was used to evaluate the goodness of fit of the logistic regression models. A *P* value above .05 indicates a good fit of the model.

## Results

### Participant Demographics

The total studied group included 249 patients (158/249, 63.5% men) aged 65.3 (SD 13.8) years. Table 1 presents basic characteristics of the studied population.

Hypertension, atherosclerosis, and heart failure were the most common diseases diagnosed in the surveyed population. More than half of the respondents (144/249, 57.8%) were hospitalized approximately twice in the previous year. Coronary angiography (146/249, 58.6%) and percutaneous coronary interventions (105/249, 42.2%) were reported as the most frequent medical procedures (Multimedia Appendix 4). In terms of health care access, approximately half of patients (125/249, 50.2%) obtained medical services at public outpatient clinics. Almost every tenth person (23/249, 9.2%) used only private clinics, while every third patient (90/249, 36.1%) scheduled follow-up visits both at private and public medical centers.

**Table 1:** Baseline socioeconomic characteristics.

Qualitative variables		Participants, n (%)
**Sex**		
	Male	158 (63.5)
	Female	91 (36.5)
**Place of residence**		
	City	211 (84.7)
	Rural	38 (15.3)
**Marital status**		
	In relationship	193 (77.5)
	Living alone	53 (21.3)
	No data	3 (1.2)
**Educational level**		
	Primary	111 (44.6)
	Secondary	75 (30.1)
	Higher	63 (25.3)
**Socioeconomic activity**		
	Retired	167 (67.1)
	Occupationally active	61 (24.5)
	Student	6 (2.4)
	No data	15 (6.0)
**Phone access**		
	Yes	246 (98.8)
	No	3 (1.2)
**Internet access**		
	Yes	158 (63.5)
	No	91 (36.5)

### Readiness for Telemedicine Solutions

The vast majority of respondents (228/249, 91.6%) remained under ambulatory follow-up care, with periodic visits to an outpatient cardiology clinic. Consistently, of the 249 total participants, 209 (83.9%) said they would accept telemedicine solutions, while 34 (13.6%) were definitely against telemedicine solutions and 6 did not respond. The results confirmed that nearly three-fourths of the respondents expressed acceptance of telemedicine to maintain contact with a physician as well as for remote monitoring of vital signs. Further details are presented in Table 2.

Furthermore, the results showed that mobile phones (167/249, 67.1%) and VT combined with provider-driven phone support (166/249, 66.7%) were the preferred communication channels between patients and doctors. Furthermore, patients' preferences regarding the envisioned form of care showed that landline phones, email contact, and webpages were chosen by 43.8% (109/249), 17.3% (43/249), and 9.2% (23/249) of participants, respectively. Of note, almost every fifth respondent chose traditional face-to-face contact with a physician (47/249, 18.9%). A total of 34 out of 249 (13.6%) patients reported that the form of contact with the doctor does not matter.

**Table 2:** Patients' readiness for telemedicine solutions.

Anticipated telemedicine services	Participants, n (%)
Remote contact with a cardiologist	176 (70.7)
Telemonitoring of vital signs (blood pressure, temperature, body weight)	168 (67.5)
Issuing e-prescriptions	161 (64.7)
Alarming health status deterioration	154 (61.8)
Scheduling and managing of medical visits	143 (57.4)
Medication reminder	106 (42.6)

### Factors Influencing Readiness for Telemedicine Solutions

Declared readiness for receiving the presented telemedical solutions significantly depended on age, gender, previous difficulties in contacting a medical doctor, and living with family (Table 3).

**Table 3:** Factors influencing patients' acceptance of telemedicine.

Independent variable		Technology accepted by patients			
		Telemedicine tools, n (%)	*P* value	Voice technology combined with provider-driven phone support, n (%)	*P* value
**Sex**			.006		.50
	Male	142 (90.4)		113 (81.9)	
	Female	66 (77.6)		52 (78.8)	
**Previous difficulties accessing cardiologist**			<.001		.003
	Yes	123 (93.9)		108 (87.8)	
	No	85 (77.9)		58 (71.6)	
**Living with family**			.004		.40
	Yes	170 (89.5)		135 (81.8)	
	No	37 (74.0)		30 (76.9)	
**Age**			.05		.20
	Younger (<68 years)	111 (90.2)		89 (84.0)	
	Older (68+ years)	98 (81.7)		77 (77.8)	
**Place of residence**			.60		.07
	City	177 (86.3)		145 (82.9)	
	Rural	31 (83.8)		20 (68.9)	
**Educational level**			.10		.05
	Primary	88 (81.5)		64 (73.6)	
	Secondary	62 (88.6)		56 (88.9)	
	Higher	57 (90.5)		46 (83.6)	
**Internet access**			.10		.30
	Yes	138 (88.5)		112 (82.9)	
	No	68 (81.9)		52 (77.6)	

Over half of patients (133/249, 53.4%) reported having encountered previous difficulties while contacting a doctor during routine clinical care management. The main reasons reported by study participants were long waiting times for a visit to an outpatient clinic (111/249, 44.6%), long waiting time in a clinic due to queue lengths (68/249, 27.3%), and substantial distance and travel time from their place of residence to a clinic (61/249, 24.5%). Results of the multivariate analysis confirmed that male sex, living with other family members, and previous difficulty of contact with physicians resulted in a willingness to use telemedicine solutions (Tables 4 and 5).

**Table 4:** Multivariate analysis between declared lack of telemedicine solution acceptance and particular independent values^a^.

Independent value (n=236)	β coefficient	OR^b^ (95% CI)
Intercept	–4.359	0.013 (0.000-0.570)
Sex (female)	–0.878	0.415 (0.183-0.943)
Age	0.009	1.009 (0.974-1.046)
Place of residence (city)	0.369	1.447 (0.487-4.304)
Level of education (primary)	–0.275	0.760 (0.443-1.304)
Living with family (yes)	0.894	2.445 (1.021-5.856)
Previous difficulties accessing cardiologist (yes)	1.364	3.913 (1.625-9.426)

^a^Model: logistic regression (logit); χ^2^=27.1; *P*<.001.

^b^OR: odds ratio.

**Table 5:** Multivariate analysis between declared lack of acceptance of voice technology combined with provider-driven phone support and particular independent values^a^.

Independent value (n=200)	β coefficient	OR^b^ (95% CI)
Intercept	–3.408	0.033 (0.001-0.877)
Sex (female)	–0.086	0.918 (0.415-2.032)
Age	–0.001	0.999 (0.971-1.027)
Place of residence (city)	0.843	2.324 (0.879-6.143)
Level of education (primary)	–0.335	0.716 (0.436-1.175)
Living with family (yes)	0.157	1.170 (0.451-3.034)
Previous difficulties accessing cardiologist (yes)	1.072	2.920 (1.377-6.192)

^a^Model: logistic regression (logit); χ^2^=13.9; *P*=.03.

^b^OR: odds ratio.

### Factors Influencing Readiness for Voice Technology

The readiness for VT was statistically significantly higher among patients with previous negative experience accessing health care (odds ratio [OR] 2.920, 95% CI 1.377-6.192) and was most frequent in patients reporting higher education and in city residents (Tables 4 and 5). The age and sex of respondents did not impact the intention to use voice agents (*P*=.20 and *P*=.50, respectively) (Table 3).

### The Null Hypothesis

The study results showed evidence against the null hypothesis, as the readiness to use telehealth services and, specifically, voice technology was associated with independent variables (Tables 3-5). Patients’ willingness to apply telemedicine was related to gender (*P*=.006), with women being less likely to declare readiness for telehealth (OR 0.415, 95% CI 0.183-0.943) than men; age (*P*=.05), with patients younger than 68 years being more likely to receive telemedicine (OR 1.009, 95% CI 0.974-1.046); living with family (*P*=.004), with individuals living with other family member declaring higher readiness for telemedicine (OR 2.445, 95% CI 1.021-5.856); and previous difficulties accessing medical care (*P*<.001), with respondents who experienced obstacles to accessing a cardiologist in the past declaring a higher intention to use telemedicine (OR 3.913, 95% CI 1.625-9.426). Place of residence and primary level of education were not associated with readiness for telehealth (*P*=.60 and *P*=.10, respectively).

Positive responses toward voice conversational agents were associated with previous difficulties accessing cardiovascular care (*P*=.003), with individuals who encountered problems obtaining medical care in the past showing higher intention to use voice technology (OR 2.920, 95% CI 1.377-6.192), and education level (*P*=.05), with respondents with a primary education being less interested in the application of conversational agents (OR 0.716, 95% CI 0.436-1.175). Place of residence appeared to be related to the respondent’s declared readiness for VT, but it did not reach statistical significance (*P*=.07). Sex, age, and living with family members were not associated with readiness for conversational agents (*P*=.50, *P*=.20, and *P*=.40, respectively).

## Discussion

### Principal Findings

This cross-sectional study evaluated factors influencing intention to use telemedical solutions. Survey results confirmed that patients with CVD declared readiness for remote health care services, including VT-based virtual care. Previous difficulties accessing cardiologists in routine clinical settings were associated with a 3 times higher likelihood of acceptance of voice assistants for medical purposes.

An increasing number of patients with CVD presents challenges to health care systems to provide ubiquitous and high-quality care [15-17]. Thus, the implementation of telehealth services may enable alternative treatment strategies [18]. Accordingly, telemedicine provides a broad spectrum of possible interventions, including self-management programs [19,20], medication adherence [21,22], monitoring of vital signs [23], medical visit reminders [24], and remote long-term follow-up [7]. However, the applicability and usefulness of new technologies must be viewed from a broader perspective that includes patients’ expectations. Of note, users’ judgments and opinions of services or products reflect dynamic changes in emotions and feelings over time. Anticipated experience refers to the expectations a person has prior to the use of a solution, while momentary experience is associated with a perception after the first interaction. As a continuum, episodic and remembered experience mirror users’ approaches toward the specific usage of a service and their general impressions after a longer period of use, respectively [25]. It is important to apply a systematic methodology while exploring patients' needs. The structured approach is a cornerstone for the creation of patient-oriented telehealth programs promoting high adoption rates [26].

This study evaluating anticipated experience indicated that a vast majority (123/249, 93.9%) of CVD patients with previous difficulties accessing traditional care declared a readiness to use telemedical solutions, including voice assistants. The survey revealed that almost half of respondents experienced delayed medical appointments (eg, long waiting times, queues) and difficulties with physical access associated with the distance between patients' homes and medical centers. In contrast, Edwards et al [27] showed that a relatively low number of patients indicated difficulty in accessing traditional health care as a reason to accept telehealth solutions.

Our study highlighted some sociodemographic variables impacting patients’ approaches toward anticipated telehealth opportunities. Men and individuals living with family reported higher interest in the use of telemedicine. Similarly, men were more likely to accept digital technologies in a study evaluating the adoption of health care apps and online tools [28]. The age of the patients has been reported to play an important role in patients' willingness to use telemedicine [29]. Despite our study showing a correlation between age and readiness for telehealth, it did not reach statistical significance, as 90.2% (111/123) of younger (<68 years) and 81.7% (98/120) of older (68+ years) respondents were interested in using telemedicine tools (*P*=.05). Similarly, a systematic review of 39 studies evaluating age-related acceptance of telemedicine showed no consistent association [30]. Moreover, a recent meta-analysis confirmed that different age groups expressed a specific level of mobile health service interest that depended on ease of use, perceived severity, and perceived vulnerability, especially for middle-aged and older individuals [31]. Interestingly, conversational agents were reported to be anticipated by younger and older adults (*P*=.20), which may help to avoid technological exclusion of older individuals. These findings are promising, as rapid progress and market penetration of voice assistants (ie, Amazon Echo, Google Home) mirror general users’ acceptance of verbal communication interfaces. In the United States, more than 87 million people were reported to use smart speakers in January 2020, which gives potential to the horizontal implementation of this novel telehealth modality [32]. Simultaneously, professional medical applications deployed using conversational agents allow patients to report health status by answering a set of clinical questions in the form of a verbal conversation between human and voice device. This trend is exemplified by the CardioCube application, which has already been tested [3] and implemented in clinical practice for remote monitoring of patients with HF and diabetes [13]. Institutionally, Nationwide Children’s Hospital in Columbus, Ohio, developed a voice service for the care coordination of children with medical complexities [11], which confirms the usability of VT in the routine management of patients.

Furthermore, in line with the findings reported by Lin et al [33], our results showed that patients living with their families showed higher intention to use the presented telehealth solutions. This observation might be associated with additional support from family members while using telehealth services.

It is important to note that patients declared different forms of preferred virtual care, and subsequent analysis showed a willingness to use mobile phones (167/249, 67.1% of respondents) as well as voice conversational agents (166/249, 66.7% of patients). Our results show that the coapplication of VT and provider-driven support through telephone contact meets patients’ expectations. These findings may help to create a basis for telehealth programs providing comprehensive real-time feedback on a patient's health status.

It must be noted that the psychological aspects related to the perception of new technologies are very complex. Our questionnaire focused on the critical elements of determining anticipated experience and readiness to use telehealth. We evaluated (1) reasons patients want to use remote care, (2) the biggest value propositions, (3) the types of patients most interested in using telehealth, and (4) the manner in which patients want to access care. However, there are more components associated with willingness and readiness that were not evaluated by our survey. Other studies have reported that the following factors should be taken into consideration when designing telemedicine programs: previous experience with technology, ability to use the technology, perceived usefulness, quality of design, and user confidence [20,28,34-36]. The last element is of special importance, as previous findings showed that patients trust telehealth services if they are provided directly by a physician [28] with the support of a case manager [33]. Furthermore, studies showed that factors associated with technology itself (performance expectancy and perceived privacy) determine older people’s intentions to receive telehealth [37], while readiness might be also facilitated by coexisiting diseases and additional costs [38].

### Study Limitations

We would like to note that we did not collect data about previous experience with telemedical solutions, which does not allow us to evaluate acceptance of virtual care. Accordingly, the study results reflected patients’ anticipated experiences and intentions toward services described by a medical doctor prior to the survey. To reduce risk of bias, we used a semistructured interview to educate study participants about each of the telehealth services included in the questionnaire. Moreover, a dedicated doctor (BK) assisted all study participants during the survey, answering additional questions regarding the clinical application of telemedicine.

The study is limited by a relatively small number of participants (N=249). However, differences in answers provided by participants had strong statistical significance supporting our findings. Moreover, the validation procedure included 30 respondents, and the e-prescription question showed the lowest Cohen score (patients’ responses should be taken with caution).

Taking into consideration that the study design was based on readiness for telemedicine, further investigations are needed to analyze acceptance of VT in clinical practice, including patients’ episodic and remembered experiences.

### Conclusion

Understanding challenges and barriers associated with the clinical use of telehealth is necessary for its successful and widespread implementation. Among many aspects, patients’ expectations and factors influencing readiness play an important role. Our study showed that intention to use VT is associated with previous difficulties accessing health care, especially in city residents and individuals who reported a higher level of education. A telehealth service combining conversational agents with provider-based phone support was anticipated by younger and older adults, which may help provide effective remote management of older individuals. Further evaluation of patients' perceptions of and incentives to use remote health care technologies is critical to designing a patient-centered solution.

## Appendix

Multimedia Appendix 1 Investigator-developed questionnaire.

Multimedia Appendix 2 Supplementary Table 1.

Multimedia Appendix 3 Semistructured interview about the clinical application of telemedicine.

Multimedia Appendix 4 Supplementary Table 2.

## Abbreviations

AI: artificial intelligence
CVD: cardiovascular disease
HF: heart failure
OR: odds ratio
VT: voice technology
