# Supplementary material for: Readiness for Voice Technology in Patients With Cardiovascular Diseases: Cross-Sectional Study
Source: J Med Internet Res. 2020 Dec 17;22(12):e20456. doi: 10.2196/20456 (PMC7775197; doi:10.2196/20456)
Supplement: Multimedia Appendix 1 [file jmir_v22i12e20456_app1.docx]

**Multimedia Appendix 1.** Investigator-developed questionnaire.

**QUESTIONNAIRE**

**‘ACCEPTANCE OF TELEMEDICAL SOLUTIONS’**

We would like to invite you to take part in a survey dedicated for patients with cardiovascular diseases. The questionnaire is anonymous and its completion will take approximately 10 minutes. We are very much interested in your opinion about the applicability and usefulness of telemedical solutions for remote contact with a cardiologist. Please mark X sign or fill out the gaps accordingly

|  |  |  |  |  |
| --- | --- | --- | --- | --- |
| 1. **Sex**: | Female |  | Male |  |

**2.** **Age:**  …………….. years

| **3.** **Place of residence:** | City |  | Rural |  |
| --- | --- | --- | --- | --- |

..…………………………………
 *(name)*

| **4. Marital status :** | Living alone |  | In relationship |  |
| --- | --- | --- | --- | --- |

| **5. Education level:** | Primary |  | Secondary |  | Higher |  |
| --- | --- | --- | --- | --- | --- | --- |
|  |  |  |  |  |  |  |

| **6. Socioeconomic   activity:** | Student |  | Occupationally   active |  | Retired |  |
| --- | --- | --- | --- | --- | --- | --- |
|  |  |  |  |  |  |  |

| **7. Phone access:** | Yes |  | No |  |
| --- | --- | --- | --- | --- |
|  | | | | |
| **8. Internet access:** | Yes |  | No |  |

**9. Have you been diagnosed with?:**

| 1. Hypertension | | Yes |  | No |  |
| --- | --- | --- | --- | --- | --- |
| 1. Diabetes | | Yes |  | No |  |
| 1. Atherosclerosis | | Yes |  | No |  |
| 1. Heart failure | | Yes |  | No |  |
| 1. Arrhythmia | | Yes |  | No |  |
| 1. Myocardial infarction | | Yes |  | No |  |
| 1. Stroke | | Yes |  | No |  |
| 1. Congenital heart disease | | Yes |  | No |  |
| 1. Other disease | *Which?…………………* | | | | |

**10. If you have been diagnosed with a cardiovascular disease, please specify how old were you at the time of diagnosis?**

………… year of life

**11. Have you ever undergone below mentioned medical procedure due to cardiovascular disease?:**

| 1. Percutaneous coronary intervention | Yes |  | No |  |
| --- | --- | --- | --- | --- |
| 1. Cardioversion | Yes |  | No |  |
| 1. Pacemaker implantation | Yes |  | No |  |
| 1. Coronary angiography | Yes |  | No |  |
| 1. Cardiac ablation | Yes |  | No |  |
| 1. Heart transplant | Yes |  | No |  |
| 1. Cardiac surgery | Yes |  | No |  |
| 1. Other procedure | Yes |  | No |  |
| *If so, please specify ……………………..* | | | | |

**12. Have you been hospitalized within last year due to your cardiovascular disease?**

| Yes |  | No |  |
| --- | --- | --- | --- |

*If so, please specify how many times have you been hospitalized with last year: …..…………*times/year

**13. Do you remain under ambulatory follow-up care based on periodic visits in outpatient cardiology clinic?**

| Yes |  | No |  |
| --- | --- | --- | --- |

*If so, please specify how many visits within a year: ..................*times/year

**14. Do you access healthcare service in cardiology outpatient clinic more than once a month?**

| Yes |  |  | No |  |
| --- | --- | --- | --- | --- |

**15. Please specify through which kind of cardiology outpatient clinic you access healthcare service:**

| Public |  | Private |  | Public and private |  |
| --- | --- | --- | --- | --- | --- |
|  |  |  |  |  |  |

**16. Have you ever experienced difficulties accessing healthcare service in cardiology outpatient clinic settings?**

| Yes |  | No |  |
| --- | --- | --- | --- |

*If so, please specify the reason:*

| 1. Long waiting time in a clinic due to queue lengths | Yes |  | No |  |
| --- | --- | --- | --- | --- |
| 1. Substantial distance/travel time from a place of  residence to a clinic | Yes |  | No |  |
| 1. Long waiting times for a visit in outpatient clinic | Yes |  | No |  |
| 1. No time due to occupational activities | Yes |  | No |  |
| 1. Financial restrictions | Yes |  | No |  |
| 1. Other | Yes |  | No |  |
| Please specify: *..............................................* | | | | |

**17. Due to the necessity for a periodic follow-up of your cardiovascular disease, would you be interested in using telemedical solutions (medical consultations at distance)?**

| Yes |  | No |  |
| --- | --- | --- | --- |

**18. If so, please specify which form of communication would you prefer when using telemedical solutions:**

| 1. Only traditional (direct, face-to-face) contact with a physician | Yes |  | No |  |
| --- | --- | --- | --- | --- |
| 1. Landline phone | Yes |  | No |  |
| 1. Mobile phone | Yes |  | No |  |
| 1. Voice technology combined with provider-driven phone support | Yes |  | No |  |
| 1. E-mail contact | Yes |  | No |  |
| 1. Web page | Yes |  | No |  |
| 1. Form of communication does not matter | Yes |  | No |  |

**19. If you accept telemedical solutions (medical consultations at distance), please specify which functionality/functionalities would you prefer:**

| 1. Remote contact with a cardiologist | Yes |  | No |  |
| --- | --- | --- | --- | --- |
| 1. Telemonitoring of vital signs (blood pressure, temperature, body weight) | Yes |  | No |  |
| 1. Issuing e-prescriptions | Yes |  | No |  |
| 1. Alarming of health status deterioration | Yes |  | No |  |
| 1. Scheduling and managing of medical visits | Yes |  | No |  |
| 1. Medication reminder | Yes |  | No |  |
| 1. Other | Yes |  | No |  |
| *Please specify …………………………………….* | | | | |
|  | | | | |
|  | | | | |
