# Supplementary material for: Readiness for Voice Technology in Patients With Cardiovascular Diseases: Cross-Sectional Study
Source: J Med Internet Res. 2020 Dec 17;22(12):e20456. doi: 10.2196/20456 (PMC7775197; doi:10.2196/20456)
Supplement: Multimedia Appendix 2 [file jmir_v22i12e20456_app2.docx]

**Supplementary Table 1*.*** Repeatability of respondents' answers to the key questions of the author's questionnaire

| Question | Kappa- Cohen  (95% CI)* | ICC  (95%CI) | Repeatability of answers [%] |
| --- | --- | --- | --- |
| Phone access | 1 | 1 | 100.0 |
| Internet access | 0.902 (0.714– 1.000) | 0.905 (0.812– 0.953) | 96.67 |
| Previous difficulties in access cardiologist | 1 | 1 | 100.0 |
| Acceptance for telemedicine tools | 1 | 1 | 100.0 |
| Patients' preferences regarding envisioned form of virtual care | | | |
| Direct contact | 1 | 1 | 100.0 |
| Landline phone | 0.792 (0.524– 1.000) | 0.799 (0.581– 0.911) | 90.91 |
| Mobile phone | 0.861 (0.597– 1.000) | 0.866 (0.709– 0.942) | 95.45 |
| Voice technology combined with provider-driven phone support | 1 | 1 | 100.0 |
| E-mail contact | 1 | 1 | 100.0 |
| Web page | 1 | 1 | 100.0 |
| Form of contact does not matter | 0.725 (0.446 – 1.000) | 0.731 (0.468– 0.875) | 86.96 |
| Patients' acceptance and readiness for telemedicine solutions | | | |
| Remote contact with a cardiologist | 0.802 (0.539-1.000) | 0.809 (0.617– 0.911) | 92.00 |
| Telemonitoring of vital signs (blood pressure, temperature, body weight) | 0.920 (0.766 – 1.000) | 0.923 (0.834– 0.956) | 96.00 |
| Issuing e-prescriptions | 0.419 (-0.004 – 0.841) | 0.434 (0.061– 0.702) | 80.00 |
| Alarming health status deterioration | 0.826 (0.596 – 1.000) | 0.833 (0.660– 0.922) | 92.00 |
| Scheduling and managing of medical visits | 0.832 (0.609 – 1.000) | 0.839 (0.666– 0.926) | 91.67 |
| Medication reminder | 0.895 (0.694 – 1.000) | 0.899 (0.783– 0.955) | 86.96 |

CI – confidence interval; ICC - interclass correlation coefficient. *The values of the Kappa-Cohen statistics with their 95% confidence interval indicate very good repeatability of answers, except for the question regarding issuing e-prescriptions (average compliance at Kappa-Cohen=0.419).
