# Supplementary material for: Readiness for Voice Technology in Patients With Cardiovascular Diseases: Cross-Sectional Study
Source: J Med Internet Res. 2020 Dec 17;22(12):e20456. doi: 10.2196/20456 (PMC7775197; doi:10.2196/20456)
Supplement: Multimedia Appendix 3 [file jmir_v22i12e20456_app3.docx]

**Multimedia Appendix 2.** Semi-structured interview about the clinical application of telemedicine.

Dear Sir/Madam,

Before you answer the survey, I would like to discuss with you potential applications of telemedical solutions to ensure you have a full understanding of the presented technologies. If you are familiar with the modality please let me know.

**1. Are you familiar with the possibility to contact your cardiologist remotely?**

**a.** **If yes** – check and go to the next question

**b.** **If no** – present the following example of implementation:

*“Instead of seeing your cardiologist in the office, you could contact him/her using phone, computer, or dedicated device. When connected remotely the doctor would evaluate your health status, give recommendations, and send you a prescription. Do you understand how remote communication works or you would like me to tell you more?”*

**2. Have you ever heard about telemonitoring of vital signs?**

**a. If yes** – check and go to the next question

**b. If no** – present the following example of implementation:

*"Routinely, blood pressure is measured by a doctor during a medical appointment in the clinic. Today, it is possible to take measurements at home and inform a doctor about results by phone or automatically through the Internet - so you do not have to call the clinic each time you check your blood pressure. The machine will send the results by itself. Do you understand how telemonitoring works or you would like me to tell you more?"*

**3. Do you know what e-prescriptions are?**

**a. If yes** – check and go to the next question

**b. If no** – present the following example of implementation:

*“Routinely, you get paper prescriptions while you see your doctor. In contrast, e-prescription can be sent to your mobile phone or email. You can use e-prescription in the pharmacy to get your drugs. Do you understand what the e-prescription is or you want me to tell you more?”*

**4. Have you heard that telemedicine can help to alarm the deterioration of your health status?**

**a. If yes** – check and go to the next question

**b. If no** – present the following example of implementation:

*“Medical device at your home can measure your health parameters like blood pressure, heart rate, and take a medical history so you speak with a device like with a person. In the case of disease, deterioration is detected, you and your doctor will be informed and you will be provided with instructions on what to do next. Do you understand how alarming works or you want me to tell you more?”*

5**. Do you know that you can schedule and manage medical visits using telemedicine?**

**a. If yes** – check and go to the next question

**b. If no** – present the following example of implementation:

*“Instead of going to the clinic to schedule, change, or cancel an appointment, you can call your doctor, use a website, mobile application, or dedicated device to do that. Do you understand how telemedicine works here or you want me to tell you more?”*

**6. Have you heard that telemedicine can remind you about medications?**

**a. If yes** – check and go to the next question

**b. If no** – present the following example of implementation:

*“Sometimes it is difficult to remember about taking medications. However, it very important for your health. Telemedicine can automatically remind you about the need to take medications at the right time. Do you understand this application of telemedicine or you want me to tell you more?”*

Now, let’s talk about possible ways of communication with your cardiologist. Again, if you are familiar with a modality let me know.

**1. Direct contact:**

**a. If yes** – check and go to the next question

**b. If no** – present the following example:

*“Each time you want to see a cardiologist you have to go to the office. There is no option to get consultation through phone or other communication channels like video-conference. Is it clear or you want me to tell you more?”*

**2. Landline phone:**

**a. If yes** – check and go to the next question

**b. If no** – present the following example:

*“To contact your cardiologist you can use a standard phone, which you have at home. Instead of going to the clinic, you will get a medical consultation during a phone call. Shall I tell you more?*

**3. Mobile phone:**

**a. If yes** – check and go to the next question

**b. If no** – present the following example:

*“You contact your cardiologist using a mobile phone. You can call, text, or use a mobile application installed on your cell phone. The doctor will provide you a medical consultation. Is it understandable or you want me to tell you more?”*

**4. Voice conversational agent**

**a. If yes** – check and go to the next question

**b. If no** – present the following example:

*“Having a voice conversational agent, you can speak with a device that understands human language. Periodically, the device will ask you a set of clinical questions and your medical history will be automatically presented to a cardiologist. The doctor will contact you by phone in case your responses need further clarification. Do you understand how voice conversational agent works or you want me to tell you more?”*

**5. E-mail contact**

**a. If yes** – check and go to the next question

**b. If no** – present the following example:

*“Instead of going to the office or calling your cardiologist, you can send him/her an email. The doctor will read it and reply. Do you understand how e-mail communication works or you want me to tell you more?”*

**6. Web page**

**a. If yes** – check and go to the questionnaire

**b. If no** – present the following example:

*“You can go to a dedicated website to check your medical results and recommendations. Moreover, using the web site you can send a message to your cardiologist who will read it and send you a message back. Do you understand how web site communication works or you want me to tell you more?”*

Thank you. Let’s now proceed to the questionnaire. If you have any questions regarding the presented telehealth solutions please let me know.
