# Supplementary material for: Readiness for Voice Technology in Patients With Cardiovascular Diseases: Cross-Sectional Study
Source: J Med Internet Res. 2020 Dec 17;22(12):e20456. doi: 10.2196/20456 (PMC7775197; doi:10.2196/20456)
Supplement: Multimedia Appendix 4 [file jmir_v22i12e20456_app4.docx]

**Supplementary Table 2.** Baseline medical characteristics.

| Diagnosed disease | Number | Frequency (%) |
| --- | --- | --- |
| **Hypertension** | 182 | 73.1 |
| **Diabetes** | 100 | 40.2 |
| **Atherosclerosis** | 122 | 49.0 |
| **Heart failure** | 106 | 42.6 |
| **Arrhythmia** | 99 | 39.7 |
| **Myocardial infarction** | 83 | 33.3 |
| **Stroke** | 18 | 7.2 |
| **Structural heart disease** | 19 | 7.6 |
| **Other diseases*** | 8 | 3.2 |
| History of previous medical procedures | | |
| **Coronary angiography** | 146 | 58.6 |
| **Percutaneous coronary intervention** | 105 | 42.2 |
| **Cardioversion** | 30 | 12.0 |
| **Pacemaker implantation** | 35 | 14.1 |
| **Cardiac ablation** | 11 | 4.4 |
| **Heart transplant** | 5 | 2.0 |
| **Cardiac surgery** | 26 | 10.4 |
| **Other procedure** | 14 | 5.6 |

*includes: pulmonary embolism, deep vein thrombosis, urolithiasis, kidney failure, hypothyroidism, cancer, gout, and ulcerative colitis.
